# Potential Impact of *COMT-rs4680 G > A* Gene Polymorphism in Coronary Artery Disease

**DOI:** 10.3390/jcdd5030038

**Published:** 2018-07-13

**Authors:** Rashid Mir, Musadiq Bhat, Jamsheed Javid, Chandan Jha, Alpana Saxena, Shaheen Banu

**Affiliations:** 1 Department of Medical Lab Technology, Faculty of Applied Medical Sciences, University of Tabuk, Tabuk 71491, Saudi Arabia; jamsheedjavidsjj@gmail.com; 2 Department of Biochemistry, Maulana Azad Medical College and Associated Hospitals, New Delhi 110002, India; bhatmusadiq@gmail.com (M.B.); rashid@ut.edu.sa (A.S.); 3 Department of Human Genetics, Punjabi University, Patiala 147002, India; chandujha58@gmail.com; 4 Department of Biochemistry, Sri Jayadeva Institute of Cardio-vascular Science & Research & Karnataka Institute of Diabetology, Bangalore 560069, India; shanbanubanu@gmail.com

**Keywords:** catechol-*O*-methyltransferase (COMT), coronary artery disease (CAD), allele specific PCR-AS-PCR, COMT Val158Met, COMT G > A

## Abstract

**Purpose**: Catechol-*O*-methyltransferase (COMT) plays a central role in DNA repair and estrogen-induced carcinogenesis. The nonsynonymous single nucleotide polymorphism (SNP) in exon 4 G > A or Val108 > 158Met or rs4680 G > A influences COMT enzyme activity. The three phenotypes of the COMT enzyme activities include COMT A/A with low enzyme activity, COMT A/G with medium enzyme activity and COMT G/G with high enzyme activity. The Met allele is associated with low enzymatic activity resulting in higher levels of prefrontal dopamine. Conversely, the Val allele is associated with high enzymatic activity and lower levels of prefrontal dopamine. The Met allele has been associated with several psychiatric disorders such as panic disorder. Many recent epidemiologic studies have investigated the association between the COMT Val158Met polymorphism and coronary artery diseases risk, but the results are inconclusive. Therefore our study was aimed to explore the association between COMT Val158Met polymorphism and the risk of coronary artery disease in India. **Methology**: This study was conducted on 100 clinically confirmed cases of coronary artery diseases and 100 healthy controls. COMT Val158Met genotyping was performed by allele-specific polymerase chain reaction (AS-PCR). **Results**: A significant correlation was observed in the COMT Val158Met genotype distribution between the coronary artery disease cases and healthy controls (*p* = 0.008). The frequencies of all three genotypes, GG, GA, AA, reported in the CAD patients were 10%, 70%, and 20%, and 30%, 60%, and 10% in the healthy controls respectively. An increased risk of coronary artery disease was observed in the codominant inheritance model for COMT-GA vs. GG genotype with an OR of 3.5, 95% CI (1.58–7.74) *p* = 0.002) and COMT-AA vs. GG genotype with an OR of 6.0 95% CI (2.11–17.3) *p* = 0.003). The higher risk of coronary artery disease was observed in the dominant inheritance model for COMT (*GA* + *AA*) vs. GG genotype (OR 3.85, 95% CI 1.76–8.4, *p* < 0.007), whereas a non-significant association was found in recessive model for COMT (*GG* + *GA* vs. *AA*) (OR = 2.01, 95% CI (0.86–4.7) *p* = 0.72). The results indicated that A allele significantly increased the risk of coronary artery disease compared to the G allele (OR = 1.8, 95% CI (1.20–2.67) *p* = 0.004). COMT Val158Met polymorphism leads to a 6.0, 3.5 and 1.8-fold increased risk of developing coronary artery disease in the Indian population and providing novel insights into the genetic etiology and underlying biology of coronary artery disease. **Conclusions**: It is concluded that COMT-AA genotype and A allele are significantly associated with an increased susceptibility to coronary artery disease in Indian population. A larger sample size can be the key to progress in establishing the genetic co-relationship of *COMT* polymorphism and cardiovascular disease.

## 1. Introduction

Coronary heart disease (CHD) has reached epidemic proportions among Indians. India is undergoing a rapid health transition with an increasing burden caused by coronary heart disease [1]. For adults over 20 years old, the estimated prevalence of CHD is around 4% in rural areas and 10% in urban areas, representing a two-fold rise in rural areas and a six-fold rise in urban areas between 1960 and 2000 [2]. Also, an increased susceptibility to CHD among Indian migrants in various parts of the world in comparison to the native population studied has been reported [3,4]. Several factors are suspected to cause high CHD morbidity and mortality rates among Indians, including genetic, metabolic, early-life, conventional, and non-conventional risk factors. However, the results from the interheart study conclusively established the role of behavioral and conventional risk factors in the prediction of CHD risk among Indians [5].

The catechol-*O*-methyltransferase is coded as the *COMT* gene and is one of the enzymes essential for metabolizing circulating serum catecholamines, catalyzes the *O*-methylation of various compounds, like catechol estrogens and dietary polyphenols, using S-adenosylmethionine (SAM) as the methyl donor. COMT also has a role in dopamine inactivation and thus is involved in the regulation of vascular resistance. Two versions of this enzyme are synthesized from the gene [6]. The longer form, called membrane-bound catechol-*O*-methyltransferase (MB-COMT), is chiefly produced by nerve cells in the brain. Other tissues, including the liver, kidneys, and blood, produce a shorter form of the enzyme called soluble catechol-*O*-methyltransferase (S-COMT). This form of the enzyme helps control the levels of certain hormones. The general function of COMT is the elimination of biologically active or toxic catechols and some other hydroxylated metabolites. During the first trimester of pregnancy, COMT present in the placenta protects the developing embryo from activated hydroxylated compounds [7,8]. COMT also acts as an enzymatic detoxicating barrier between the blood and other tissues, shielding against the detrimental effects of xenobiotics. COMT may serve some unique or indirect functions in the kidney and intestinal tract by modulating the dopaminergic tone. The same may be true in the brain, where only one single gene COMT codes for both S-COMT and MB-COMT using two separate promoters. Both rat and human S-COMTs contain 221 amino acids, and their molecular weights are 24.8 and 24.4 kD, respectively. Rat MB-COMT contains 43 and human MB-COMT contains 50 additional amino acids, of which 17 (rat) and 20 (human) are hydrophobic membrane anchors [9,10].

The remainder of the MB-COMT molecule is suspended on the cytoplasmic side of the intracellular membrane. The enzyme coding region contains a well-investigated single-nucleotide-polymorphism (SNP) in codon 158 (rs4680), in which valine (Val) is substituted to methionine (Met) through a substitution of guanine to adenine. This common functional A/G polymorphism in the *COMT* gene produces three genotype groups (homozygousVal158Val, heterozygous Val158Met, and homozygous Met158Met), which result in different enzyme activities based on changes in thermostability [11,12] as depicted in Table 1. The three phenotypes of the COMT enzyme activities include COMT A/A with low enzyme activity, COMT A/G with medium enzyme activity and COMT G/G with high enzyme activity. The Met allele is associated with low enzymatic activity resulting in higher levels of prefrontal dopamine. Conversely, the Val allele is associated with high enzymatic activity and lower levels of prefrontal dopamine. The Met allele has been associated with several psychiatric disorders such as panic disorder [13,14].

COMT-Val158Met-polymorphism has been intensively studied. Associations have been found with the susceptibility and appearance of cognitive phenotypes, psychiatric disorders, changes in brain activation and structure, and cancer susceptibility [15,16]. Furthermore, links between COMT genotypes and the increased risk of coronary events or the outcome of patients with ischemic heart disease were revealed [17,18]. COMT Val158Met polymorphism is associated with the increased risk of acute coronary events and it may interact with high serum tHcy levels [19]. Several small population-based studies found genetic variation in *COMT* to be associated with coronary heart disease [20] and hypertension in men [21,22,23]. Based on study findings, the Val/Val genotype appears to be associated with a higher prevalence of increased systolic blood pressure compared with the Met/Met or Met/Val genotypes at the *COMT* gene [24]. In another study, the low activity COMT genotype (Met158Met) was associated with heavy alcohol use [25]. The influence of COMT G158A polymorphism was studied in some human cancers, such as breast [26], endometrial [27], and prostate carcinomas [28]. The unifying hypothesis is that hyperhomocysteinemia may exert its pathogenic effects largely through metabolic accumulation of S-adenosyl-l-homocysteine (SAH)a potent non-competitive inhibitor of COMT-mediated methylation metabolism of various catechol substrates. Therefore, we wanted to test the hypothesis that the functional polymorphism Val108Met in the *COMT* gene could modify the risk of a coronary event in our population.

## 2. Materials and Methods

### 2.1. Study Population

This study was a population-based cohort study designed to investigate risk factors for CAD related to outcomes in middle-aged men and women from India. A total of 200 blood samples were analyzed among which 100 were from CAD patients (96 men and 4 women) and 100 healthy controls.

### 2.2. Patient Selection Criteria 

#### 2.2.1. Inclusion Criteria

Patients undergoing elective angiography for the evaluation of stable chest pain at the Sri Jayadeva Institute of Cardiovascular Science and Research and Karnataka Institute of Diabetology, Bangalore were recruited. Some non-invasive tests were performed including an electrocardiogram (ECG or EKG), ambulatory electrocardiography, Holter monitoring, chest X-ray, echocardiogram (echo), cardiac computed tomography (CCT), exercise stress test and Myocardial Perfusion Imaging (MPI) or Multigated acquisition scan (MUGA). Patients were not selected on the basis of the chest pain characteristics (i.e., whether or not suspicious of angina or myocardial ischemia), but merely that chest pain was a presenting symptom prompting an elective angiography. The cohort was classified based on their coronary angiographic findings as either significant CAD (stenosis ≥ 50%) or ICAD (no stenosis or stenosis < 50%). The exclusion criteria included patients with a history of non-coronary cardiac disorders cases, previously performed coronary bypass surgery, or percutaneous transluminal coronary angioplasty (PTCA) due to their treated coronary status.

#### 2.2.2. Selection Criteria of Healthy Controls

A healthy control cohort was established from participants visiting for routine checkup to Sri Jayadeva Institute of Cardiovascular Science and Research and Karnataka Institute of Diabetology, Bangalore. These participants completed the informed consent form and questionnaire. The healthy control cohort was selected based on self-reported absence of previous heart attack or angina. Some biochemistry tests were also performed. The exclusion criteria included participants with available cardiac history, history of angina and/or myocardial infarction.

### 2.3. Sample Collection

From each patient, a 4 mL peripheral blood sample was collected in EDTA vials after obtaining informed consent from all patients. The samples were collected only after the approval letter was obtained from the concerned Institutional Ethics Committee.

### 2.4. Measurement of Serum Lipids and Apolipoproteins

After overnight fasting and prior to coronary angiography, blood was collected from each subject. Serum total cholesterol, triglyceride, high density lipoprotein (HDL), and low density lipoprotein (LDL)-C concentrations and total cholesterol/HDL-C ratios were determined using the standard method.

### 2.5. Data Collection

The corresponding data from each patient were collected and analyzed for their cholesterol level, diabetes, RBS, LDL, HDL, TGL, and smoking. The patient follow-up was performed regularly and samples were collected on a regular basis.

### 2.6. Genotyping Analysis

#### 2.6.1. DNA Extraction

Genomic DNA was extracted using a Blood DNA Isolation Kit (GEB100) from Geneaid (Taipei, Taiwan) from the whole blood according to manufacturer’s instructions. The mean optical density (OD) of the DNA samples was determined as 1.8 nm using Nanodrop.

#### 2.6.2. AS-PCR for COMT (Val158Met) gene Polymorphism

The enzyme coding region of COMT contains a well-investigated single nucleotide polymorphism (SNP) (rs4680), with the presence of guanine or adenine at nucleotide 475, encoding a valine (Val) or methionine (Met) at codon 158, producing three genotype groups: homozygous Val158Val or GG, heterozygous Val158Met or G/A, and homozygous Met158Met or AA. The same primers were used for detecting COMT 475A > G gene polymorphism as shown in Table 2.

**Figure 1:**
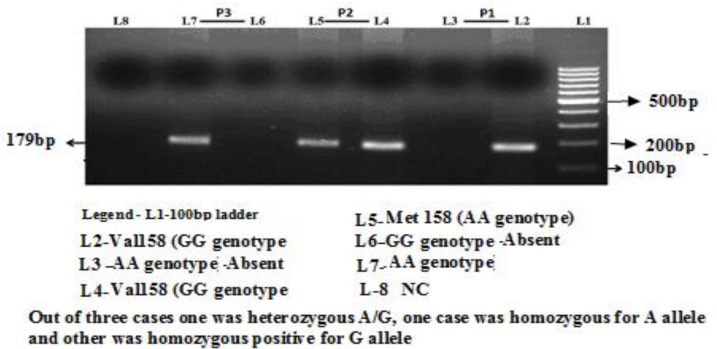
Allele-specific polymerase chain reaction (PCR) for the detection of catechol-*O*-methyltransferase (COMT) (Vall58Met) G > A gene polymorphism.

COMT (Val158Met) G > A gene polymorphism was identified by AS-PCR. The amplification was accomplished with a 25-μL reaction mixture containing 100 ng template DNA, 0.25 μL of 25 pmol of each primer, 2.5 μL 10 mM dNTP’s 1.5 μL of 20 mM MgCl_2_, and 0.3 μL of 5 U/μL Taq polymerase with 2.5 μL of 10X Taq Buffer (Fermantas, Waltham, MA, USA). The thermocycling program involved initial denaturation at 96 °C for 10 min and 30 cycles: 96 °C for 30 s, 56 °C for 45 s, 72 °C for 45 s, and final extension at 72 °C for 5 min. Presence or absence of each allele (PCR products) were observed by electrophoresis on 2% agarose gel. After electrophoresis, amplified products were visualized by ethidium bromide stain using ultraviolet trans-illumination as depicted in Figure 1. Genotyping was performed without the knowledge of the case/control status of the study subjects.

### 2.7. Stastical Analysis

CAD patients and controls were compared using statistical analysis performed using the SPSS 16.0 software package. Chi-square analysis and Fisher exact test were used to compare COMT rs4680 G > A gene polymorphism frequency with several clinical aspects, including sex, age, cholesterol, HDL, LDL, TGL, hypertension, diabetes, alcohol, Pan Masala, and smoking. Hardy-Weinberg equilibrium was tested with a χ^2^ test to compare the observed genotype frequencies within the case-control groups. The *p* value was considered to be significant when <0.05.

## 3. Results

### 3.1. Baseline Characteristics of the Study Subjects

All demographic features and clinicopathological characteristics of CAD patients are depicted in Table 3. At the time of analysis, a total of 100 coronary artery disease patients (96 men and 4 women) and 100 healthy controls were included in the study. Both age and sex distribution of both cohorts were not significantly different. Out of 100 cases, 53 were older than 50 years and 47 were 50 years old or younger (*p* = 0.089) as depicted in Table 3. Of the CAD cases, 55, 93, 91, 79, and 51% patients had RBS, cholesterol, HDL, LDL, and TGL below or equal to 140, 200, 40, 100, and 150 mg, respectively, whereas 45, 7, 9, 21, and 49% of patients had levels greater than 140, 200, 40, 100, and 150 mg of RBS, cholesterol, HDL, LDL, and TGL, respectively. The clinical status of patients showed that 25% and 32% were positive and 75% and 68% were negative for hypertension and type 2 diabetes (T2D), respectively. Also, 57% and 35% of subjects were smokers and alcoholics, respectively, and 43% and 65% subjects were non-addictive (Table 3).

#### 3.1.1. Case Control Genotype Distribution

A significant difference was observed in genotype distribution among the CAD cases and healthy controls (*p* = 0.008) as depicted in Table 4. The frequencies of the Val/Val, Val/Met, and Met/Met genotypes in coronary artery disease patients were 10, 70, and 20%, respectively. The frequencies of Val/Val, Val/Met, and Met/Met genotypes in healthy controls were 30, 60, and 10%, respectively. The distribution of the COMT genotypes was consistent with the Hardy-Weinberg equilibrium. A higher frequency of heterozygosity (G/A) was reported in CAD patients compared with the healthy controls. Moreover, the frequency of the A allele (fA) was found to be significantly higher among patients than healthy controls (0.55 vs. 0.40). The frequency of the G allele (fG) was found to be significantly lower in patients than in healthy controls (0.45 vs. 0.60).

#### 3.1.2. Case Control Genotype Distribution

Clinical correlation of genotype frequency of *COMT* polymorphism was determined with respect to all clinicopathological characteristics of CAD patients. A significant correlation was found between age (*p* = 0.04), random blood sugar (RBS) (*p* = 0.016), and triglyceride concentration (*p* = 0.006) as depicted in the Table 5. A non-significant correlation was found with HDL, low density lipoprotein (LDL), and cholesterol. A significant correlation between COMT gene polymorphism in CAD cases with respect to T2D but not hypertension was also found. With respect to different habits, a significant association with alcohol consumption was reported but a non-significant association was reported with smoking and eating pan masala.

### 3.2. Association of COMT rs4680 G > A Gene Variation with Coronary Artery Disease

A multivariate analysis based on logistic regression, including odds ratio (OR), risk ratio (RR), and risk difference with 95% confidence intervals (CI), were calculated for each group to estimate the association between the COMT rs4680 G > A polymorphism and the risk of CAD in Indian patients (Table 6). The odds and risk ratios with 95% confidence intervals were calculated for each group to estimate the degree of association between the COMT rs4680 G > A variant and the risk of CAD risk in Indian patients. Our findings showed that COMT rs4680 G > A polymorphism is associated with an increased risk of coronary artery disease for COMT-GA vs. GG genotype (OR = 3.5, 95% CI 1.58–7.74; RR 1.62, 95% CI 1.25–2.10; *p* = 0.0002) in the codominant inheritance model. Similarly, a significant correlation was found for COMT-AA vs. GG genotype with (OR 6.0 (2.11–17.3), RR 2.25 (1.31–3.8), *p* = 0.003) in the codominant inheritance model. An increased risk of coronary artery disease was found in the dominant inheritance model for COMT-(GA + AA) vs. GG genotype (OR 3.85, 95% CI 1.76–8.4, *p* < 0.007), whereas a non-significant association was reported in the recessive model for COMT-(GG + GA vs. AA) (OR = 2.01, 95% CI 0.86–4.7, *p* = 0.72). The A allele significantly increased the risk of coronary artery disease (OR 1.8, 95% CI 1.20–2.67, *p* = 0.004) compared to the G allele. Therefore, we determined that COMT rs4680 G > A polymorphism leads to a 6.0-, 3.5-, and 1.8-fold increase in the risk of developing coronary artery disease in Indian populations. Results indicated that COMT rs4680 G > A polymorphism is associated with susceptibility to coronary artery disease, providing novel insights into the genetic etiology and underlying biology of this disease.

## 4. Discussion

Catechol-*O*-methyltransferase is one of several enzymes that degrade catecholamines such as dopamine, epinephrine, and norepinephrine. As the regulation of catecholamines is impaired in a number of medical conditions, several pharmaceutical drugs target COMT to alter its activity and therefore the availability of catecholamines. Levodopa, a precursor of catecholamines, is an important substrate of COMT. COMT inhibitors, like entacapone, save levodopa from COMT and prolong the action of levodopa. Entacapone is a widely used adjunct drug with levodopa therapy. When given with an inhibitor of dopa decarboxylase (carbidopa or benserazide), levodopa is optimally saved. This “triple therapy” is becoming a standard in the treatment of Parkinson’s disease [29].

The enzyme coding region contains a well-investigated single-nucleotide-polymorphism (SNP) in codon 158 (rs4680), in which valine (Val) is substituted by methionine (Met) through a substitution of guanine to adenine, which subsequently leads to different enzyme activities based on changes in thermostability. The Met/Met-genotype has a three- to four-fold reduced enzymatic activity compared with the Val/Val-genotype The Val variant catabolizes dopamine at up to four times the rate of its methionine counterpart [6].

COMT-Val158Met-polymorphism has been intensively studied. Associations have been found with the susceptibility and appearance of cognitive phenotypes, psychiatric disorders, changes in brain activation and structure, and cancer susceptibility. Furthermore, links between COMT genotypes and the increased risk of coronary events or the outcome of patients with ischemic heart disease have been revealed [8].

The main finding of our study is that common functional Val158Met polymorphism of the *COMT* gene is an independent risk factor for coronary artery disease events in the Indian population, which may also interact with serum haemocystein, alcohol, and triglycerides to further increase the risk of coronary events. Men who were homozygous (Met/Met-genotype) for the low activity allele of COMT had increased serum levels of estradiol, which has shown that the altered estrogen status could be involved in this effect. In that study, 74% of study subjects were men and the interaction between COMT genotype and homocysteine were not studied [26,27]. Opposite results were reported, in which the low activity genotype of COMT reduced the risk of myocardial infarction.

The possible mechanism behind the protective effect of the low activity COMT genotype on myocardial infarction is not fully understood. However, altered estrogen status could be involved in the protective effect of this genotype, since the low activity COMT genotype less efficiently removes estrogens, which, as a consequence, could lead to more cardiovascular protection from estrogen. Not estrogen itself but its hydroxylated metabolites are thought to at least partially mediate the cardiovascular protection provided by estrogen [27]. Thus, increased levels of estrogen and/or the hydroxylated metabolites might be involved in the cardioprotective effect of the low activity COMT genotype.

The cardioprotective effect of low activity COMT genotype was most evident in older patients [30]. The reason for this could be that the level of estrogen might be sufficient to provide cardiovascular protection independent of COMT activity at younger ages. However, with increasing age, the levels decline and approach a critical level, where the low activity COMT enzyme results in protection by slowing down the degradation of estrogen and its metabolites [31]. Previous studies showed that type I alcoholism is more common among subjects with the low activity COMT genotype (LL), compared with high activity (HH) or heterozygotic (LH) genotypes. Alcoholism and heavy smoking are highly comorbid and are cotransmitted in the Indian population. Catechol-*O*-methyltransferase (COMT) functional polymorphism, Val158Met, has been associated with alcoholism in Caucasians [32]. Our study investigated the association of alcohol, pan masala consumption, and tobacco use with Val158Met polymorphism and results indicated that the low activity COMT genotype may contribute significantly to alcohol intake in the general male population.

Our results require confirmation in an independent population of patients and controls. The frequency of COMT low activity genotype was higher among the subjects with abnormal RBS and diabetes. Association of the *COMT* gene with diabetes and nephropathy has been reported in a study conducted on an Asian Indian population in which a genetic variant showed an association with diabetic nephropathy [33]. An important strength of the present study is that it is a retrospective study with very well characterized subjects and cardiovascular endpoints. Strikingly, the most prominent risk factors for CAD were hypertension, diabetes mellitus, and metabolic syndrome, which are associated with a reduced antioxidant capacity and increased oxidative stress, resulting in endothelial dysfunction, crucial and early events in atherogenesis. Endothelial dysfunction is a major player in the development and progression of vascular pathology in pulmonary arterial hypertension. The alteration of the shear forces alters the biology of the endothelial monolayer and subsequently the susceptibility of vessels to atherosclerosis. Patients with intracranial atherosclerotic disease (ICAD) ICAD often have coexisting systemic atherosclerosis and multiple potential stroke mechanisms that affect their prognosis, suggesting that extensive evaluations of overlapping diseases may allow better risk stratification [34]. Ion channels that are directly implicated in the development of atherosclerosis have not been identified. This functional polymorphism results in the met variant having a 3–4 fold lower enzymatic activity than the val variant, and is, therefore, inversely correlated with endogenous levels of dopamine and other COMT substrates, both at rest and with exercise or cardiac surgery-induced stress. In addition, our population-based case control study has reported that this genetic variation in *COMT* is associated with increased risk of coronary heart disease. 

## 5. Conclusions

Our findings indicated that common *COMT* polymorphism is associated with an increased risk of coronary artery disease events. Also the results indicated that the COMT-AA genotype and A allele are significantly associated with an increased susceptibility to coronary artery disease. A larger sample size could be key to establishing the genetic co-relation of *COMT* polymorphism and cardiovascular disease.

## Figures and Tables

**Table 1:** Catechol-*O*-methyltransferase COMT enzyme and its three phenotypes activities (COMT-475G > A or rs4680 G > A or Met158Val gene polymorphism).

Homozygous COMT G/G	Homozygous Val158val	Enzyme with high activity	Low level of dopamine
Heterozygous COMT A/G	Heterozygous Val158Met	Enzyme with medium activity	Medium level of dopamine
Homozygous COMT A/A	Homozygous Met158met	Enzyme with low activity	High level of dopamine

**Table 2:** Allele-specific polymerase chain reaction (AS-PCR) primers for *COMT* (Val158Met) genotyping.

Primer Direction	Nucleotide Change	Amino Acid Change	Primer Sequence	Annealing Tempt	Product Size
Forward F			5′-ACTGTGGCTACTCAGCTGTG-3′	56 °C	169 bp
Reverse R1	G allele	(Val158)	5′-GCATGCACACCTTGTCCTT-3′		
Reverse R2	A allele	(Met158)	5′-GCATGCACCACCTTGTCCTTCAT-3′		

**Table 3:** Clinicopathological characteristics of coronary artery disease (CAD) patients and controls.

Variable	No. of CAD Cases	Healthy Controls
Subjects	100 100%	100 100%
Men	96 96%	90 90%
Women	4 4%	10 10%
**Age difference**		
Age ≤ 50	47 47%	60 60%
Age > 50	53 53%	40 40%
**Variable for CAD cases**	**Number**	**(%)**
**Random Blood Sugar**		
≤140 mg	55	57%
>140 mg	45	43%
**Cholesterol**		
≤200 mg	93	93%
>200 mg	7	7%
**HDL**		
≤40 mg	91	91%
>40 mg	9	9%
**LDL**		
≤100 mg	79	79%
>100 mg	21	21%
**TGL**		
≤150 mg	51	51%
>150 mg	49	49%
**Hypertension**		
Yes	14	14%
No	86	86%
**Type 2 Diabetes**		
Yes	23	23%
No	77	77%
**Smoking**		
Yes	63	63%
No	37	37%
**Alcohol**		
Yes	40	40%
No	60	60%
**Pan Masala**		
Yes	2	2%
No	98	98%

**Table 4:** Genotype and allele frequency of *COMT* polymorphism in coronary artery disease (CAD) patients and controls.

Subjects	Number	G/G (Val/Val)	A/G (Val/Met)	A/A (Met/Met)	X^2^	df	G	A	*p* Value
**Patients**	100	10 (10%)	70 (70%)	20 (20%)	14.1	2	0.45	0.55	<0.008
**Controls**	100	30 (30%)	60 (60%)	10 (10%)			0.60	0.40	

**Table 5:** Clinical correlation of *COMT* polymorphism with respect to clinicopathological characteristics of CAD patients and controls.

Allele/Genotype	Number	G/G (Val/Val)	A/G (Val/Met)	A/A (Met/Met)	*p* Value
**COMT G > A correlation with sex**					
Men	96	09	68	19	0.53
Women	4	01	02	01	
**COMT G > A correlation with age**					
≤50 years	47	03	27	17	0.0480
>50 years	53	07	43	03	
**COMT G > A correlation with RBS**					
≤140 mg	**55**	03	45	7	0.016
>140 mg	**45**	07	25	13	
**COMT G > A correlation with cholesterol**					
≤200 mg	93	09	69	15	NS
>200 mg	07	01	01	05	
**COMT G > A correlation with HDL**					
≤40 mg	91	08	65	18	NS
>40 mg	9	02	05	02	
**COMT G > A correlation with LDL**					
≤100 mg	79	07	58	14	NS
>100 mg	21	03	12	06	
**COMT G > A correlation with TGL**					
≤150 mg	51	08	40	03	<0.006
>150 mg	49	02	30	17	
**Correlation with hypertension**					
Hypertension	14	02	08	04	NS
No hypertension	86	08	62	16	
**Correlation with diabetes**					
Diabetes	23	04	13	06	0.023
No Diabetes	77	06	57	14	
**Correlation with smoking**					
Smoking	63	05	45	13	<0.667
No Smoking	37	05	25	07	
**Correlation with alcohol consumption**					
Alcohol	40	04	24	16	<0.001
No Alcohol	60	06	46	04	
**Correlation with Pan Masala consumption**					
Pan Masala	02	1	1	0	<0.206
No Pan Masala	98	09	69	20	

**Table 6:** Association of COMT rs4680 G/A gene variation with coronary artery disease.

Genotype	Healthy Controls		CAD Patients		OR (95% CI)	Risk Ratio (RR)	*p*-Value
	**(*N* = 100)**	**%**	**(*N* = 100)**	**%**			
**Codominant inheritance model**							
COMT-GG	30	30%	10	10%	1 (ref.)	1 (ref.)	
COMT-GA	60	60%	70	70%	3.5 (1.58–7.74)	1.62 (1.25–2.10)	0.002
COMT-AA	10	10%	20	20%	6.0 (2.11–17.03)	2.25 (1.31–3.8)	<0.003
**Dominant inheritance model**							
COMT-GG	30	30%	10	10%	1 (ref.)	1 (ref.)	
COMT-(*GA* + *AA*)	70	70%	90	90%	3.85 (1.76–8.4)	1.71 (1.33–2.20)	<0.0007
**Recessive inheritance model**							
COMT-(*GG* + *GA*)	90	90%	80	81.63%	1 (ref.)	1 (ref.)	
COMT-AA	10	10%	18	18.36%	2.02 (0.86–4.7)	1.05 (0.74–1.51)	0.72
**Allele**							
COMT-G	120	60%	90	45.45%	1 (ref.)	1 (ref.)	
COMT-*A*	80	40%	108	54.55%	1.8 (1.20–2.67)	1.34 (1.09–1.64)	<0.004

## References

[1] Garwood S. (2010). Cardiac surgery-associated acute renal injury: New paradigms and innovative therapies. J. Cardiothorac. Vasc. Anesth..

[2] Scanlon P.D., Raymond F.A., Weinshilboum R.M. (1979). Catechol-*O*-Methyltransferase: Thermolabile enzyme in erythrocytes of subjects homozygous for allele for low activity. Science.

[3] Volavka J., Bilder R., Nolan K. (2004). Catecholamines and aggression: The role of COMT and MAO polymorphisms. Ann. N. Y. Acad. Sci..

[4] Yadav S., Singhal N.K., Singh V., Rastogi N., Srivastava P.K., Singh M.P. (2009). Association of single nucleotide polymorphisms in CYP1B1 and COMT genes with breast cancer susceptibility in Indian women. Dis. Mark..

[5] Silbiger J.J., Stein R., Trost B., Shaffer J., Kim J.H., Cohen P., Kamran M. (2012). Coronary angiographic findings and conventional coronary artery disease risk factors of Indo-Guyanese immigrants with stable angina pectoris and acute coronary syndromes. Ethn. Dis..

[6] Meloto C.B., Segall S.K., Smith S., Parisien M., Shabalina S.A., Rizzatti-Barbosa C.M. (2015). *COMT* gene locus: New functional variants. Pain.

[7] Blair C., Sulik M., Willoughby M., Mills-Koonce R., Petrill S., Bartlett C., Greenberg M. (2015). Family Life Project Investigators. Catechol-*O*-methyltransferase Val158met polymorphism interacts with early experience to predict executive functions in early childhood. Dev. Psychobiol..

[8] Mannisto P.T., Kaakkola S. (1999). Catechol-*O*-methyltransferase (COMT): Biochemistry, molecular biology, pharmacology, and clinical efficacy of the new selective COMT inhibitors. Pharmacol. Rev..

[9] He Z., Sun X., Guo Z., Zhang J.H. (2011). Expression and role of COMT in a rat subarachnoid hemorrhage model. Acta Neurochir. Suppl..

[10] Walton E., Liu J., Hass J., White T., Scholz M., Roessner V., Gollub R., Calhoun V.D., Ehrlich S. (2014). MB-COMT promoter DNA methylation is associated with working-memory processing in schizophrenia patients and healthy controls. Epigenetics.

[11] Chen J., Lipska B.K., Halim N., Ma Q.D., Matsumoto M., Melhem S. (2004). Functional analysis of genetic variation in catechol-*O*-methyltransferase (COMT): Effects on mRNA, protein, and enzyme activity in postmortem human brain. Am. J. Hum. Genet..

[12] Detrait E.R., Carr G.V., Weinberger D.R., Lamberty Y. (2016). Brain catechol-*O*-methyltransferase (COMT) inhibition by tolcapone counteracts recognition memory deficits in normal and chronic phencyclidine-treated rats and in COMT-Val transgenic mice. Behav. Pharmacol..

[13] Lachman H.M., Papolos D.F., Saito T., Yu Y.M., Szumlanski C.L., Weinshilboum R.M. (1996). Human catechol-*O*-methyltransferase pharmacogenetics: Description of a functional polymorphism and its potential application to neuropsychiatric disorders. Pharmacogenetics.

[14] Zhu B.T. (2002). Catechol-*O*-Methyltransferase (*COMT*)-mediated methylation metabolism of endogenous bioactive catechols and modulation by endobiotics and xenobiotics: Importance in pathophysiology and pathogenesis. Curr. Drug Metab..

[15] Minassian A., Young J.W., Geyer M.A., Kelsoe J.R., Perry W. (2018). The COMT Val158Met Polymorphism and Exploratory Behavior in Bipolar Mania. Mol. Neuropsychiatry.

[16] Zhou Q., Wang Y., Chen A., Tao Y., Song H., Li W., Tao J., Zuo M. (2015). Association between the COMT Val158Met polymorphism and risk of cancer: evidence from 99 case-control studies. Onco Targets Ther..

[17] Happonen P., Voutilainen S., Tuomainen T.P., Salonen J.T. (2006). Catechol-*O*-Methyltransferase gene polymorphism modifies the effect of coffee intake on incidence of acute coronary events. PLoS ONE.

[18] Suzuki K., Nakazato H., Matsui H., Koike H., Okugi H., Kashiwagi B. (2003). Genetic polymorphisms of estrogen receptor alpha, CYP19, catechol-*O*-methyltransferase are associated with familial prostate carcinoma risk in a Japanese population. Cancer.

[19] Voutilainen S., Tuomainen T.P., Korhonen M., Mursu J., Virtanen J.K., Happonen P., Alfthan G., Erlund I., North K.E., Mosher M.J. (2007). Functional COMT Val158Met polymorphism, risk of acute coronary events and serum homocysteine: The Kuopio ischaemic heart disease risk factor study. PLoS ONE.

[20] Hall K.T., Nelson C.P., Davis R.B., Buring J.E., Kirsch I., Mittleman M.A., Loscalzo J., Samani N.J., Ridker P.M., Kaptchuk T.J. (2014). Polymorphisms in catechol-*O*-methyltransferase modify treatment effects of aspirin on risk of cardiovascular disease. Arterioscler. Thromb. Vasc. Biol..

[21] Miyaki K., Htun N.C., Song Y., Ikeda S., Muramatsu M., Shimbo T. (2012). The combined impact of 12 common variants on hypertension in japanese men, considering gwas results. J. Hum. Hypertens..

[22] Htun N.C., Miyaki K., Song Y., Ikeda S., Shimbo T., Muramatsu M. (2011). Association of the catechol-*O*-methyl transferase gene Val158Met polymorphism with blood pressure and prevalence of hypertension: Interaction with dietary energy intake. Am. J. Hypertens..

[23] Bastos P., Gomes T., Ribeiro L. (2017). Catechol-*O*-Methyltransferase (COMT): An Update on Its Role in Cancer, Neurological and Cardiovascular Diseases. Rev. Physiol. Biochem. Pharmacol..

[24] Hagen K., Pettersen E., Stovner L.J., Skorpen F., Holmen J., Zwart J.A. (2007). High systolic blood pressure is associated with Val/Val genotype in the catechol-*O*-methyltransferase gene. The Nord-Trøndelag Health Study (HUNT). Am. J. Hypertens..

[25] Zhu B.T. (2002). On the mechanism of homocysteine pathophysiology and pathogenesis: A unifying hypothesis. Histol. Histopathol..

[26] Wedrén S., Rudqvist T.R., Granath F., Weiderpass E., Ingelman-Sundberg M., Persson I. (2003). Catechol-*O*-methyltransferase gene polymorphism and post-menopausal breast cancer risk. Carcinogenesis.

[27] Doherty J.A., Weiss N.S., Freeman R.J., Dightman D.A., Thornton P.J., Houck J.R. (2005). Genetic factors in catechol estrogen metabolism in relation to the risk of endometrial cancer. Cancer Epidemiol. Biomark. Prev..

[28] Chen Y., Yu X., Li T., Yan H., Mo Z. (2016). Significant association of catechol-*O*-methyltransferase Val158Met polymorphism with bladder cancer instead of prostate and kidney cancer. Int. J. Biol. Mark..

[29] Henchcliffe C., Waters C. (2002). Entacapone in the management of Parkinson’s disease. Expert Opin. Pharmacother..

[30] Brown A.L., Lane J., Holyoak C., Nicol B., Mayes A.E., Dadd T. (2011). Health effects of green tea catechins in overweight and obese men: A randomized controlled cross-over trial. Br. J. Nutr..

[31] Lohoff F.W., Weller A.E., Bloch P.J., Nall A.H., Ferraro T.N., Kampman K.M., Pettinati H.M., Oslin D.W., Dackis C.A., O’brien C.P. (2008). Association between the catechol-*O*-methyltransferase (COMT) Val158Met polymorphism and cocaine dependence. Neuropsychopharmacology.

[32] Enoch M.A., Waheed J.F., Harris C.R., Albaugh B., Goldman D. (2006). Sex differences in the influence of COMT Val158Met on alcoholism and smoking in plains American Indians. Alcohol. Clin. Exp. Res..

[33] Prasad P., Kumar K.M., Ammini A.C., Gupta A., Gupta R., Thelma B.K. (2008). Association of dopaminergic pathway gene polymorphisms with chronic renal insufficiency among Asian Indians with type-2 diabetes. BMC Genet..

[34] Hoshino T., Sissani L., Labreuche J., Ducrocq G., Lavallée P.C., Meseguer E., Guidoux C., Cabrejo L., Hobeanu C., Gongora-Rivera F. (2018). AMISTAD Investigators. Prevalence of Systemic Atherosclerosis Burdens and Overlapping Stroke Etiologies and Their Associations with Long-term Vascular Prognosis in Stroke with Intracranial Atherosclerotic Disease. JAMA Neurol..

